# The Response of Dark Septate Endophytes (DSE) to Heavy Metals in Pure Culture

**DOI:** 10.1371/journal.pone.0047968

**Published:** 2012-10-31

**Authors:** Yihui Ban, Ming Tang, Hui Chen, Zhouying Xu, Haihan Zhang, Yurong Yang

**Affiliations:** 1 College of Life Sciences, Northwest A & F University, Yangling, Shaanxi, China; 2 College of Forestry, Northwest A & F University, Yangling, Shaanxi, China; 3 School of Environmental and Municipal Engineering, Xi'an University of Architecture and Technology, Xi'an, Shannxi, China; Dowling College, United States of America

## Abstract

Dark septate endophytes (DSE) occur widely in association with plants exposed to heavy metal stress. However, little is known about the response of DSE exposed to heavy metals. In this study, five DSE were isolated from the roots of *Astragalus adsurgens* Pall. seedlings growing on lead-zinc mine tailings in China. Based on morphological characteristics and DNA sequence analyses, the isolates were identified as *Gaeumannomyces cylindrosporus*, *Paraphoma chrysanthemicola*, *Phialophora mustea*, *Exophiala salmonis*, and *Cladosporium cladosporioides*. *G*. *cylindrosporus* was selected to explore responses to Pb stress. Scanning electron microscopic observations of *G*. *cylindrosporus* grown on solid medium revealed curling of hyphae and formation of hyphal coils in response to Pb. In contrast, in liquid medium, hyphae became thick and swollen with an increase in Pb (II) concentration. We interpret that these changes are related to the variation in cell wall components. We also demonstrated that fungal melanin content increased with the addition of Pb(II). Melanin, as an important component in the cell wall, is known to be an essential antioxidant responsible for decreasing heavy metal toxicity. We also measured the total soluble protein content and glutathione (GSH) concentrations in *G*. *cylindrosporus* and found that they initially increased and then decreased with the increase of Pb(II) concentrations. The antioxidant enzyme activities were also examined, and the results showed that superoxide dismutase (SOD) activity was significantly positively correlated with Pb(II) concentrations (r = 0.957, P<0.001). Collectively, our observations indicate that the intracellular antioxidant systems, especially fungal melanin, play an important role in abating the hazards of heavy metals.

## Introduction

Soil pollution with heavy metals has become one of the most serious worldwide environmental problems [1]. This problem has attracted considerable public attention, because the continued increase of metal levels in soil poses a health risk to humans and animals through the food chain or contaminated drinking water [2]. Endophytic fungi not only have ability to protect against heavy metal toxicity but also increase nutrient acquisition of host plants and enhance their metabolic activity to combat stress [3], [4]. A wide range of fungi from all major taxonomic groups have been found in heavy metal polluted soil, and some of them have evolved resistance to heavy metals [5]. Thus, the research on mechanisms that protect endophytic fungi against heavy metal toxicity needs to be further conducted.

Dark septate endophytes (DSE), which are one of groups of endophytic fungi, are ubiquitous in various stressful environmental conditions [6]. They are conidial or sterile ascomycetous fungi that colonize living plant roots without causing any apparent negative effects [7]. DSE can colonize nearly 600 plant species representing about 320 genera and 114 families [6], [7]. Many dominant plant species in heavy metal contaminated land are widely associated with DSE fungi [8]. Thus, these fungi may play an important role in protecting plants from heavy metal stress [9]. Likar and Regvar [10] found that the DSE colonization of *Salix caprea* L. showed good correspondence with soil Pb and Cd and concluded that DSE could improve metal tolerance of willows to high heavy metal contamination. Deram et al. [11] reported that DSE colonization was constant when Cd concentrations in soil increased compared to the disappearance of AM colonization, which indicated that soil heavy metals are toxic to AM but not to DSE. DSE fungi can be readily isolated from heavy metal contaminated sites. Zhang et al. [12] isolated three strains of DSE from a waste smelter site in southwest China, and found that the tolerance of the DSE strains varied between metal species and strains. Zhan et al. [13] demonstrated that melanin content in *Exophiala pisciphila* increased with the addition Cd(II) to the medium over the range of 50 to 350 mg/L.

Melanin in DSE hyphae was deemed to be the most important component of the cell wall that decreases heavy metal toxicity [8], [14]. Several studies shown that fungal melanin has a capacity to bind heavy metal ions [15]. However, the understanding of the role of fungal melanin in heavy metal tolerance of DSE is still lacking. The alteration of fungal melanin will directly affect mycelial morphology [16], and the mycelial morphology and characteristics of hyphae are closely related to the presence of heavy metals [17]. Glutathione (GSH) is another important heavy metal tolerance agent. It is the most abundant cellular thiol-rich heavy metal binding peptide in fungi [18], and recent works tend to consider the soluble tripeptide as the first line of defense against heavy metal cytotoxicity [19]. Less in known about the role of antioxidant enzymes such as superoxide dismutase (SOD) and catalase (CAT) in heavy metal tolerance even though their activities vary considerably in response to heavy metal stress [20], [21]. The induction of antioxidant enzymes, especially SOD, is an important protection mechanism to decrease oxidative damage under heavy metal stress, which plays a key role in cellular defense mechanisms against reactive oxygen species (ROS) [22].

The aims of this work were to (1) characterize DSE fungi isolated from the roots of plants grown in heavy metal contaminated soil, (2) measure the changes in hyphal morphology of DSE fungi under different Pb(II) concentrations stress, and (3) study the responses of antioxidant substances in DSE fungi, such as melanin, GSH, SOD, and CAT, to Pb(II) stress.

## Materials and Methods

### Ethics statement

The sampling area is not privately-owned or protected in any way, so no specific permits were required for the described field studies. The field studies did not involve endangered or protected species. The fungal species used in the experiment were isolated from lead-zinc mine tailings by ourselves. Informed consent was obtained from all participants.

### Isolation and morphological identification of the DSE species

The five DSE isolates were all isolated from the roots of *Astragalus adsurgens* Pall. that grew naturally on Qiandongshan lead-zinc mine tailings, Fengxian county, Shaanxi province, China (106°38′E, 33°49′N; elevation: 1189 m). The total concentrations of Pb, Zn, and Cu in the soil were 1350.7, 2105.4, and 526.4 mg/kg, respectively. *A. adsurgens* was the dominant plant species on the mine tailings, and the DSE root colonization we determined was 68% according to the method of McGonigle et al. [23]. The sampling was performed in September 2010. Root samples were collected from five randomly selected individuals of *A. adsurgens* that grew naturally on the mine tailings. DSE fungi were isolated according to the protocol of Ahlich and Sieber [24] with slight modifications. Roots were washed under running tap water, aseptically cut into 2.0–2.5 cm long pieces, surface-sterilized in 99% ethanol for 1 min, 35% hydrogen peroxide for 5 min, 99% ethanol for 30 s, and washed three times with deionized water. A segment of 2–3 mm length was aseptically excised from the middle of each root piece and incubated at 25°C in the dark on potato dextrose agar (PDA) in 90 mm Petri dishes. Dark-pigmented fungi that emerged from the roots were isolated and transferred to fresh PDA agar plates. After 2 weeks of incubation, the morphology of hyphae and spores was observed under a light microscope (Olympus, BX51).

### DNA extraction, PCR amplification, and sequencing

DNA was extracted from 2-week-old pure cultures of the five DSE species by the CTAB method [25]. Primers ITS1 (5′-TCCGTAGGTGAACCTGCGC-3′) and ITS4 (5′-TCCTCCGCTTATTGATATGC-3′) [26] were used for amplification of the DSE fungal rDNA internal transcribed spacer (ITS) regions. The PCR reactions were performed in a S1000 Thermal Cycler (Bio-Rad, USA). PCR was performed in 50 µl reaction volumes containing 2 µl genomic DNA, 2 µl of each primer (10 µM), 5 µl 10× PCR buffer, 7 µl 25 mM Mg^2+^, 2 µl 2.5 mM dNTP, 1 µl Taq polymerase, and 29 µl ddH_2_O. The conditions included an initial denaturation at 94°C for 3 min, followed by 35 cycles of 94°C for 1 min, 55°C for 45 s, and 72°C for 2 min, and a final extension at 72°C for 8 min. PCR products were separated in a 1.0% agarose gel, stained with ethidium bromide and bands were visualized under UV light. Expected bands were excised and purified with E.Z.N.A.® Gel extraction kit (Omega Bio-Tek, Inc., Norcross, GA, USA). The purified PCR products were ligated into the pGEM-T Easy vector (Promega, Madison, WI, USA), and *Escherichia coli* DH5α competent cells were transformed with the ligation products according to manufacturer's recommended protocol. Reconfirmed clones were used for sequencing (Nanjing GenScript Corporation, China) using the universal primers SP6 and T7. All DNA sequences were edited and compared to the available sequences from the National Center for Biotechnology Information (NCBI) using the basic local alignment search tool (BLAST) [27], and were submitted to GenBank under the accession numbers JN123358–JN123361 and JF508361.

### Sequence alignment and phylogenetic analysis

For the construction of the phylogenetic tree, sequence alignment was carried out using ClustalW [28], and phylogenetic analyses were conducted with MEGA 5.0 [29] using the neighbour-joining method with the Kimura two-parameter distance measure. Confidence values were estimated from bootstrap analysis of 1000 replicates [30].

### Resynthesis with host plant

In order to verify that the five isolates were DSE fungi, resynthesis experiments were performed in *A*. *adsurgens* using the method of Wu and Guo [31] with modifications. Seeds of *A*. *adsurgens* were surface-sterilized in 70% ethanol for 50 s, 0.1% HgCl_2_ for 7 min, and rinsed three times in deionized water. The seeds of *A*. *adsurgens* were then aseptically planted onto Murashige and Skoog (MS) solid medium in culture bottles. The well-growing seedlings were aseptically transferred to the culture bottles containing autoclave-sterilized 40 ml vermiculite-litter (10∶1) and 12 ml MS liquid medium. Inoculum of DSE fungi was added as two 5-mm plugs excised from an edge of an actively growing colony on PDA. Fungus-free controls were mock-inoculated with sterile PDA plugs. All cultures were carried out in a growth chamber with a photoperiod of 12 h per day at 25°C.

### DSE colonization

The roots of *A*. *adsurgens* were collected after 6 weeks of cultivation. The roots from the same treatment were randomly selected and samples from different parts of the root system were examined. Root samples were cleared and stained by the method of Koske and Gemma [32]. The roots were cut into 1-cm pieces, cleared in 2.5% KOH at 90°C for 60 min, acidified with 5 N HCl and stained with 0.05% trypan blue in lactoglycerol. The stained roots were mounted on glass slides and observed under a light microscope (Olympus, BX51) with a digital camera (Qimaging, MicroPublisher 5.0 RTV). Pictures showing melanized septate hyphae and microsclerotia were managed by Image-Pro Express 6.0 analyses software (Olympus).

### Screening and selection of heavy metal-resistant DSE fungi

The sensitivity of the isolates to heavy metal ions was evaluated by the minimum inhibitory concentration (MIC) and the effective concentration that inhibits 50% of mycelial growth (EC_50_). Modified Melin-Norkrans (MMN) medium [33] was selected as the basal medium in order to have maximal metal availability and to avoid metal precipitation. 5-mm diameter plugs were excised from an edge of actively growing 2-week-old colonies and placed on solid MMN medium amended with heavy metal ions. The concentrations used were: 0 to 4.5 mg/ml for Pb(II) and Zn(II), and 0 to 2.0 mg/ml for Cu(II). Colonies were harvested after they were incubated in the dark for 2 weeks, and the agar was melted by microwave heating for 1 min. All mycelia were taken out carefully, rinsed three times with distilled water at 60°C, dried to a constant weight at 80°C, and weighed [34]. Liquid medium offers several advantages over agar culture. However, as DSE fungi are root endophytes, it is important to note that the differentiation of fungal mycelia in the absence of a solid substrate may affect the sensitivity to metals [35].

### Observation of mycelial morphology under Pb(II) stress by scanning electron microscopy

Two different kinds of hyphae were observed. One was cultured on solid MMN medium with different concentrations of Pb(II) for two weeks, and the other was grown in liquid MMN medium for one week. However, the treatment methods of two different hyphae samples were similar. The mycelium was separated off and treated with 2.5% of glutaral solution at 4°C. After being rinsed with phosphate buffer (50 mM; pH 6.8) for 2 h, the samples were dehydrated using a series of increasing concentrations (30, 50, 70, 85, 90, 95, 100%) of ethanol solution. The process of drying at critical point, mounting, and gold spraying were completed last. Then the samples were observed and photographed using JSM-6360LV SEM (JEOL, Japan).

### Extraction and purification of melanin

According to the method described by Ellis and Griffiths [36], the mycelial mass was homogenized in KOH (1 M) using a homogenizer at high speed for 10 min. The homogenate was then sonicated in an ultraturax processor (SONICS, VCX-130). The melanin was extracted from the hyphae with hot alkali (1 M KOH at 100°C for 5 h) under reflux in an atmosphere of nitrogen. After filtration, the filtrate was acidified with HCl (3 M) until precipitation at pH 2.0. The resulting black precipitate was collected by centrifugation (10,000×*g* for 15 min) and washed with distilled water. The crude melanin was purified by an acid hydrolysis with HCl (7 M) at 100°C for 2 h. The non-hydrolysable residues were collected by centrifugation (10,000×*g* for 20 min) and then successively washed with HCl (0.01 M) and distilled water. The black precipitate was dried in a desiccator and kept under nitrogen until needed.

### Soluble protein, GSH content determination

The protein content was measured using a bovine serum albumin (BSA) protein Assay Kit (Jiancheng, Nanjing, China) with BSA as standard [37].

GSH was measured enzymatically in the presence of NADPH and 5,5′-dithiobis-2-itrobenzoic acid (DTNB). The mycelium was harvested after being inoculated in MMN liquid medium with different concentrations of heavy metals for 2 weeks. Harvested mycelium was rinsed three times with distilled water and dabbed dry between filter paper. After being crushed in liquid N_2_, the mycelium (50 mg) was homogenized in 1.5 ml of 0.1 N HCl [38]. The GSH assay mixture contained 1.0 ml supernatant, 2.5 ml 100 mmol/L phosphate buffer (pH 7.7), and 0.2 ml 0.6 mmol/L DTNB. The reduction of DTNB to nitrothiobenzoate was determined spectrophotometrically at 412 nm for 90 s following Anderson's [39] description. The amount of GSH was determined from a calibration curve and a sample blank lacking GSH was used to determine the background rate.

### Measuring the antioxidant enzyme activities in the hyphae of *G*. *cylindrosporus* under Pb(II) stress

Enzyme activities of SOD and CAT were measured with a commercial kit (Jiancheng, Nanjing, China) according to its manual instructions. SOD activity was measured through the inhibition of nitroblue tetrazolium (NBT) reduction by O_2_^−^ generated by the xanthine/xanthine oxidase system [40]. One SOD activity unit was defined as the amount of enzyme that inhibits the rate of NBT reduction by 50%. CAT activity was measured by the decomposition of hydrogen peroxide by the method of Aebi [41]. One CAT activity unit was defined as the degradation of 1 mmol H_2_O_2_ per mg protein for one second. The SOD and CAT activities were expressed as U/mg protein.

### Statistical analysis

The experiments were set up with four replicates. Data were statistically analyzed using SPSS version 16.0 software package (SPSS Inc., USA) and presented as means ± standard errors (SE). Duncan's post-hoc pair-wise comparisons were used to test the significance of differences between treatments, and results were considered significant at P<0.05. Graphical work was carried out using SigmaPlot for Windows version 10.0 software packages.

## Results

### Morphological and molecular identification of DSE species

**Figure 1:**
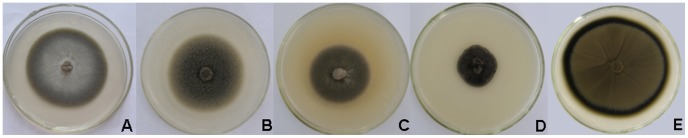
Two-week-old colonies of the five isolates on PDA. Two-week-old colonies of the five isolates on PDA. A, *Gaeumannomyces cylindrosporus* (B145); B, *Paraphoma chrysanthemicola* (B100); C, *Phialophora mustea* (BC42); D, *Exophiala salmonis* (BC5); E, *Cladosporium cladosporioides* (B142).

**Figure 2:**
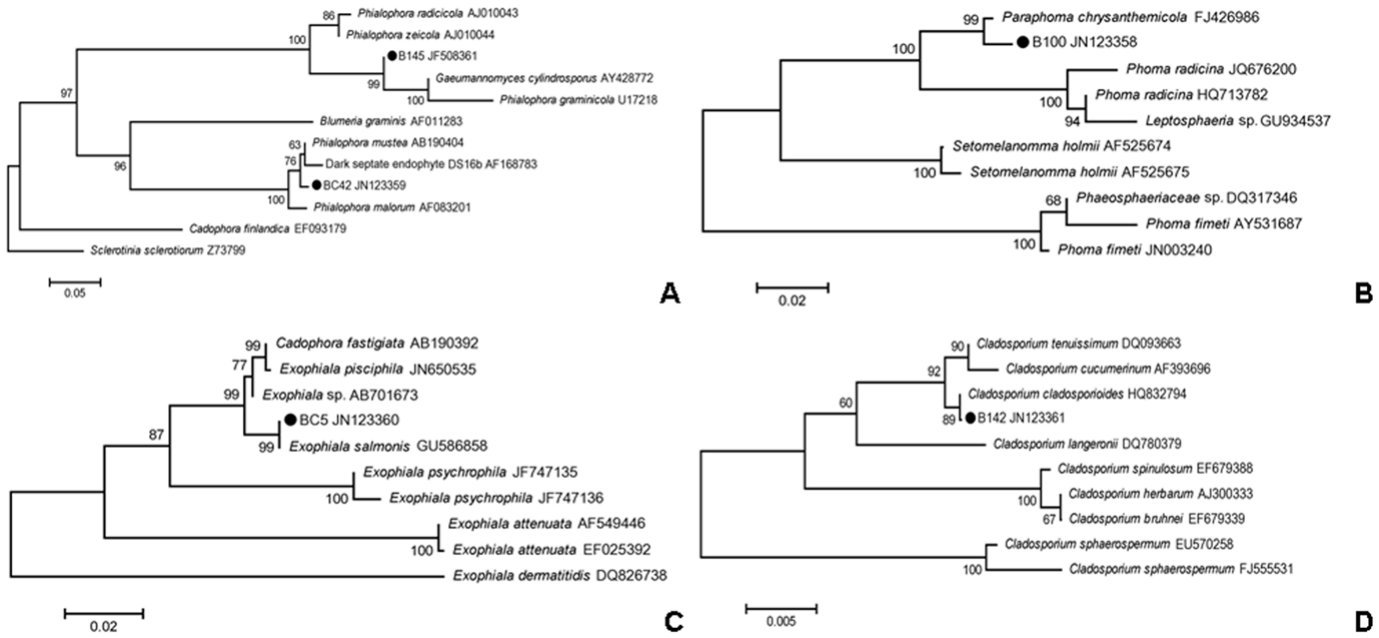
Neighbor-joining phylogenetic trees showing the placement of B145 and BC42 (A), B100(B), BC5(C), and B142(D) based on ITS1-5.8S-ITS2 sequences. The Kimura two-parameter model was used for pairwise distance measurement. Numbers on branches were values generated from 1000 bootstrap replicates. Bootstrap values of 50% were shown above branch nodes. • denoted the isolates.

Five DSE isolates (B145, B100, BC42, BC5, and B142) have been obtained from the roots of *A*. *adsurgens* and their colony morphology is shown in Fig. 1. All the five DSE fungi produced dark colonies under cultivation conditions on PDA medium and formed either round or elliptical colonies. Hyphae of these DSE fungi were dark, septate, and about 3–8 µm wide. The colony of B145 grew slowly and its center was gray (Fig. 1A). The hyphae were almost straight, septate, and slight brown. The colony of B100 was greyish-brown and powdery (Fig. 1B), the hyphae were septate and darkly pigmented. The colony of BC42 with septate and mostly brown hyphae, grew slowly, black, and often became brown with age (Fig. 1C). The diameter of slow growing BC5 colony was only about 3 cm after 2 weeks of incubation (Fig. 1D); the colony was dark-brown and downy, and the hyphae were septate and pale brown. The colony of B142 was mostly olivaceous-brown and powdery (Fig. 1E), and the hyphae were septate and pigmented. Only B142 formed conidia under cultivation conditions, while the other four DSE fungi were absent of sporulation. Conidia of B142 were smooth, echinulate, and produced in branched acropetal chains. The ITS1-5.8S rDNA-ITS2 region sequence data of the five isolates were deposited to GenBank with accession numbers of JF508361 (B145), JN123358 (B100), JN123359 (BC42), JN123360 (BC5), and JN123361 (B142). Based on the results of the phylogenetic analysis, B145, *G*. *cylindrosporus* (AY428772), and *P*. *graminicola* (U17218) were clustered together with a bootstrap value of 99% (Fig. 2A). B145 had a very close evolutional relationship with *G*. *cylindrosporus* (AY428772) (with sequence identities of 99%). BC42, *P*. *mustea* (AB190404), and dark septate endophyte DS16b (AF168783) formed a cluster with moderate support (76%) (Fig. 2A). BC42 sequence matched the ITS region of *P*. *mustea* (AB190404) with 98% sequence identities. B100 and *P*. *chrysanthemicola* (FJ426986) formed a cluster of 99% (Fig. 2B). BC5 and *E*. *salmonis* (GU586858) formed a monophyletic clade with 99% bootstrap support and 100% sequence identities (Fig. 2C). B142 and *C*. *cladosporioides* (HQ832794) formed a terminal cluster with 89% bootstrap support (Fig. 2D). In conclusion, the five isolates were different DSE species, belonging to different taxa: *G*. *cylindrosporus* (B145), *P*. *chrysanthemicola* (B100), *P*. *mustea* (BC42), *E*. *salmonis* (BC5), and *C*. *cladosporioides* (B142).

### Colonization of DSE fungi in the roots of *A*. *adsurgens*

**Figure 3:**
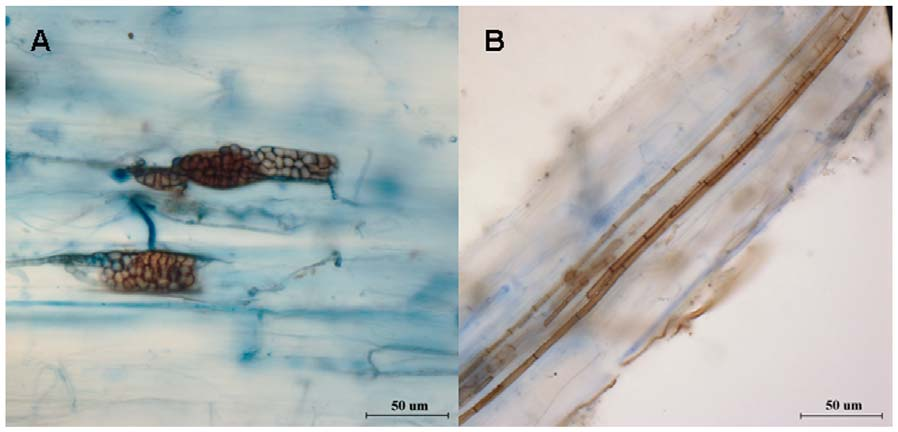
Typical structures of DSE fungi colonized in the roots of *A*. *adsurgens* seedlings. A, Structure of microsclerotia formed by *P*. *chrysanthemicola*; B, Intercellular melanized septate hyphae formed by *G*. *cylindrosporus*.

All the five isolates produced melanized septate hyphae and microsclerotia in root cortical cells of inoculated *A*. *adsurgens* seedlings, and the typical DSE structures are shown in Fig. 3. Hyphae of mature microsclerotia (Fig. 3A) were septate, thick walled, and packed tightly within host cells. The dark hyphae grew along the cortex parallel to the longitudinal axis of the roots (Fig. 3B).

### Screening of heavy metal-resistant DSE fungi

**Figure 4:**
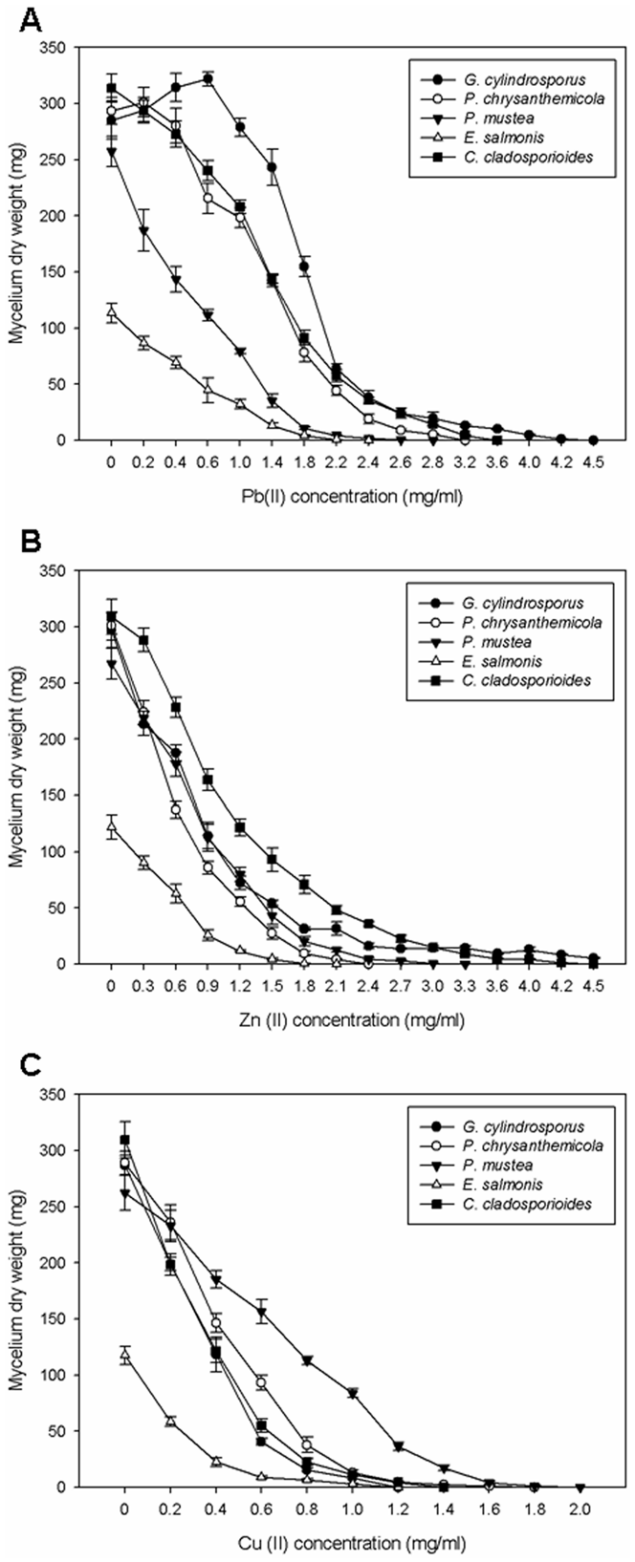
The biomass of the five DSE fungi treated with different concentrations of Pb(II). The biomass of the five DSE fungi grown on solid MMN medium amended with different concentrations of Pb(II), Zn (II), and Cu(II) for two weeks. Data are means ± SE (n = 4).

The EC_50_ and MICs of the three metal ions for the studied DSE species are shown in Table 1. Most of the five DSE fungi have high tolerance to Pb(II) and Zn(II) as compared to their tolerance to Cu(II). Pb(II) appeared less toxic in comparison with the other two metal ions for *G*. *cylindrosporus*, *P*. *chrysanthemicola*, and *C*. *cladosporioides*. Their MIC values to Pb(II) were over 3.0 mg/ml. *G*. *cylindrosporus* showed the highest MIC value of 4.5 mg/ml. Their EC_50_ values were over 1.0 mg/ml and the EC_50_ value to Pb(II) of *G*. *cylindrosporus* was 1.83 mg/ml which is higher than the others. At low Pb(II) concentrations (0.2 mg/ml, 0.4 mg/ml, and 0.6 mg/ml), *G*. *cylindrosporus* was very resistant and exhibited strong growth, already exceeding the control in biomass (Fig. 4A). Therefore, the Pb(II) tolerance of *G*. *cylindrosporus* was selected for the following experiments.

**Table 1:** The MIC and EC_50_ values of Pb(II), Zn(II), and Cu(II) for the DSE fungi.

DSE species	EC_50_ (mg/ml)			MIC (mg/ml)		
	Pb(II)	Zn(II)	Cu(II)	Pb(II)	Zn(II)	Cu(II)
*G*. *cylindrosporus*	1.83	0.64	0.30	4.5	4.6	1.2
*P*. *chrysanthemicola*	1.12	0.54	0.39	3.2	2.4	1.8
*P*. *mustea*	0.45	0.72	0.60	2.8	3.3	2.0
*E*. *salmonis*	0.46	0.52	0.17	2.4	2.1	1.2
*C*. *cladosporioides*	1.03	0.97	0.29	3.6	4.5	1.4

MIC is defined as the minimum inhibitory concentration of heavy metal that completely inhibited the growth of test fungi; EC_50_ is the effective concentration of heavy metal that inhibits 50% of mycelial growth.

### Morphological changes of *G*. *cylindrosporus* under Pb(II) stress

**Figure 5:**
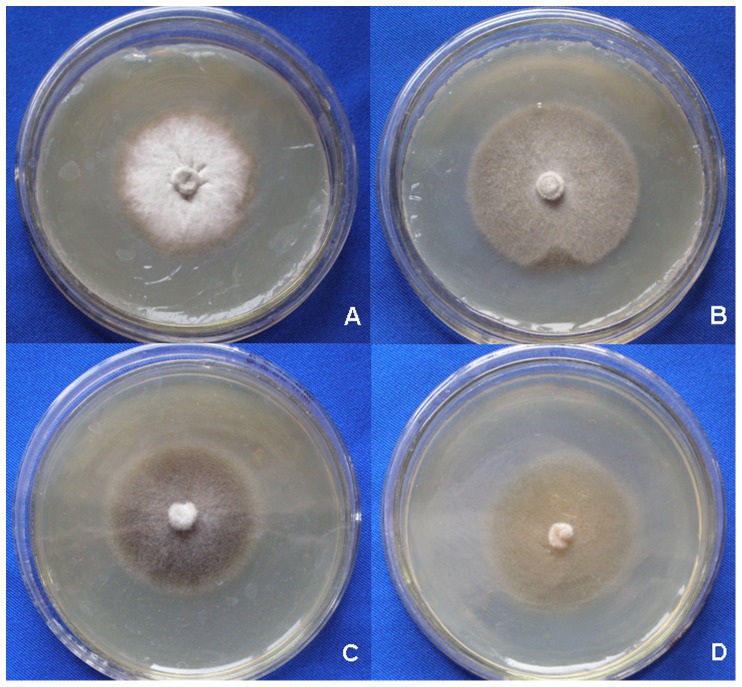
Colony morphology of *G*. *cylindrosporus* exposed to different concentrations of Pb(II). Colonies of *G*. *cylindrosporus* on MMN medium with different concentrations of Pb(II) for 2 weeks. A, Colony of *G*. *cylindrosporus* with no Pb(II) stress; B, Colony of *G*. *cylindrosporus* on MMN medium supplemented with 0.2 mg/ml of Pb(II); C, Colony of *G*. *cylindrosporus* on MMN medium supplemented with 0.6 mg/ml of Pb(II); D, Colony morphology of *G*. *cylindrosporus* on MMN medium supplemented with 1.0 mg/ml of Pb(II).

The morphology of colonies and hyphae of *G*. *cylindrosporus* were observed when cultured on MMN medium with different concentrations of Pb(II) for 2 weeks. There were remarkable changes in colony morphology in the presence of different heavy metal concentrations compared to the heavy metal-free control (Fig. 5). All colonies turned dark or brown on agar medium under Pb(II) concentrations of 0.2 mg/ml, 0.6 mg/ml, and 1.0 mg/ml (Fig. 5B, C, and D). With the increase of Pb(II) concentrations,the colony color changed from brown to black (Fig. 5B and C) and then became pale yellow when *G*. *cylindrosporus* was cultured under Pb(II) concentration of 1.0 mg/ml.

**Figure 6:**
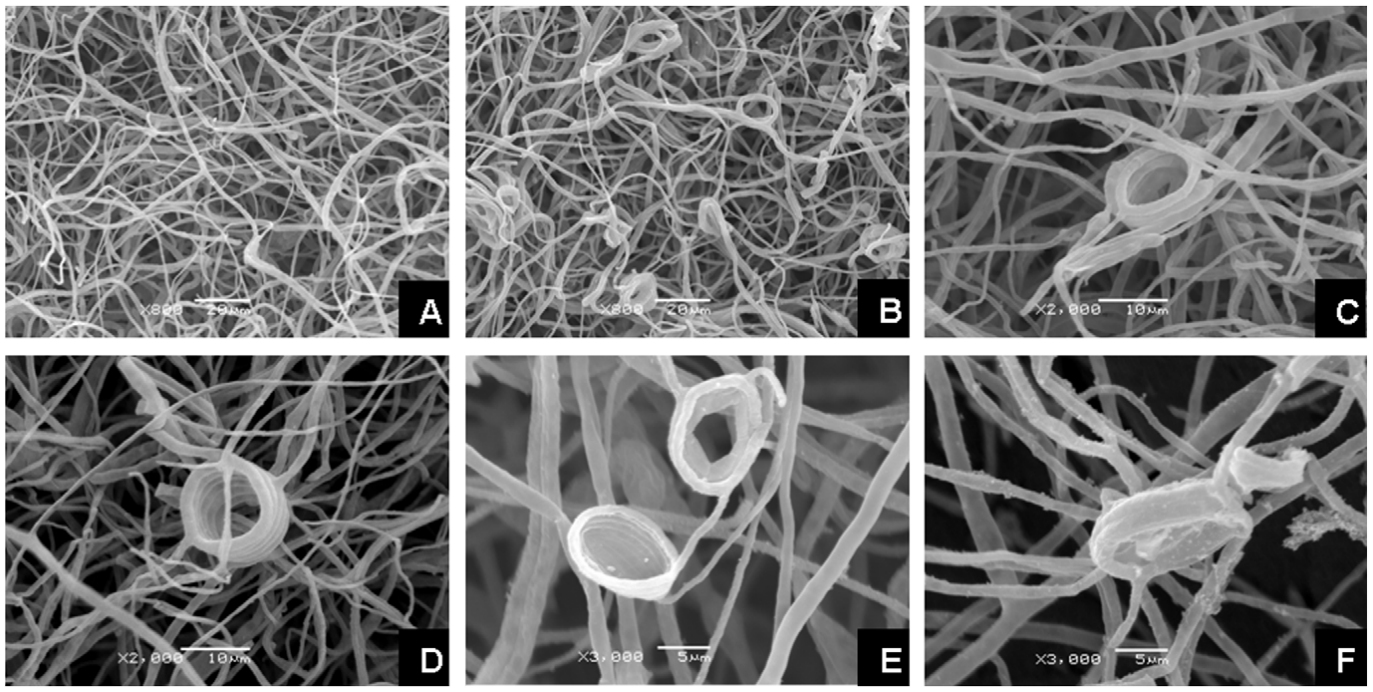
Hyphal morphology of *G*. *cylindrosporus* cultured on solid medium. Scanning electron micrographs of the hyphae from the colony edge of *G*. *cylindrosporus*. Twisting and looping of individual hyphae and formation of intertwined hyphal strands occurred when Pb(II) was added in the medium. However, there was no obvious relationship between the number of hyphal coils or the extent of hyphal twisting and the concentrations of Pb(II). A, Hyphae from the untreated control; B, Hyphae from a colony treated with 0.2 mg/ml Pb(II); C–F, Mycelial special morphology of *G*. *cylindrosporus* under Pb(II) stress.

**Figure 7:**
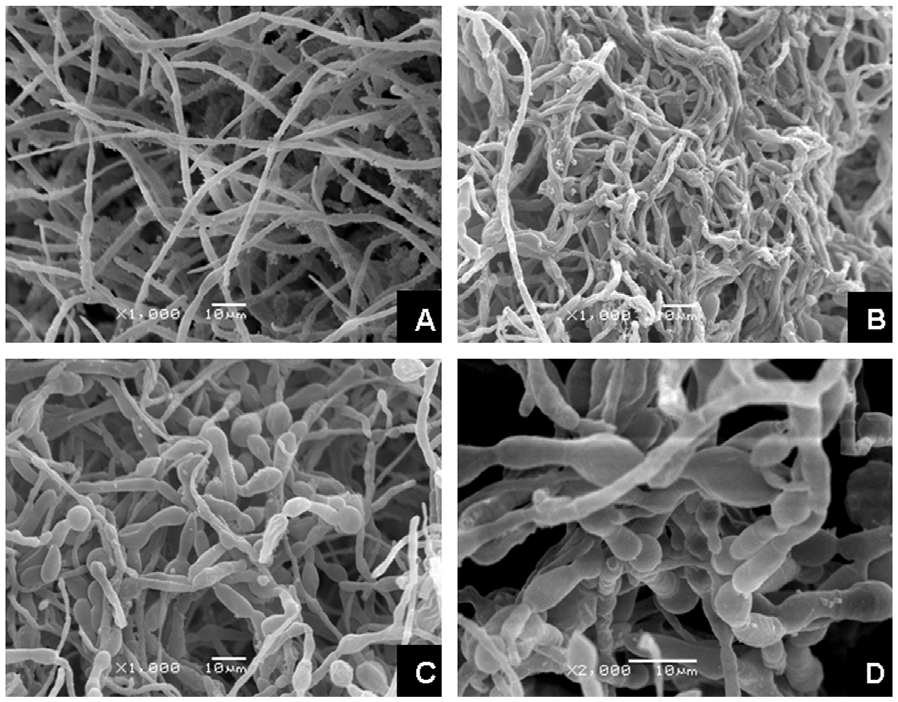
Hyphal morphology of *G*. *cylindrosporus* cultured in liquid medium. Scanning electron micrographs of the hyphae of *G*. *cylindrosporus* cultivated for a week in liquid medium supplemented with different concentrations of Pb(II). A–D, Mycelial morphology of *G*. *cylindrosporus* treated with 0, 0.1, 0.3, and 0.5 mg/ml Pb(II), respectively.

Changes in mycelial morphology were observed with the scanning electronic microscope (SEM). Pb(II) caused substantial changes in hyphal morphology (Fig. 6). In contrast to the mycelial morphology of the control (Fig. 6A), there was twisting and looping of individual hyphae and formation of intertwined hyphal strands under Pb(II) stress (Fig. 6B). The stressed hyphae also formed regular, tight, and spiral hyphal loops (Fig. 6C–F). The mycelial morphology of *G*. *cylindrosporus* grown in liquid medium under different concentrations of Pb(II) was also observed. The morphological changes were also obvious. There were remarkable differences in hyphal morphology between the two different growing conditions. When Pb(II) concentration was low (0.1 mg/ml), hyphae curled and twisted without a change in diameter of hyphae (Fig. 7B). However, when Pb(II) concentration was increased to 0.3 mg/ml, hyphae obviously became thick and swollen (Fig. 7C). This morphological change was more remarkable when the concentration was higher (0.5 mg/ml) (Fig. 7D).

### Changes of antioxidant substances in the hyphae of *G*. *cylindrosporus* under Pb(II) stress

**Figure 8:**
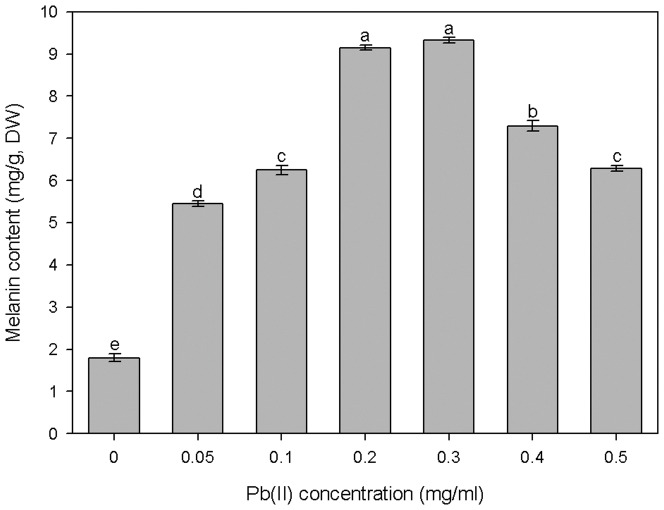
Melanin content in *G*. *cylindrosporus* under different Pb(II) concentrations stress. The influence of Pb(II) on melanin content in *G*. *cylindrosporus* under liquid culture condition. DW represented dry weight. Different letters above bars indicate significant differences (P<0.05) assessed by Duncan's test. Data are means ± SE (n = 4).

The contents of melanin, soluble protein, and GSH and the activities of SOD and CAT in *G*. *cylindrosporus* were measured to detect the responses of antioxidant substances to Pb(II) stress. Fig. 8 shows that Pb(II) treatments caused a significant increase in melanin content. When exposed to 0.2 and 0.3 mg/ml Pb(II), melanin content in *G*. *cylindrosporus* increased to 508% and 518% of the control, respectively. Though melanin content declined somewhat in the 0.4 and 0.5 mg/ml Pb(II) treatments, the respective measurements were still elevated to 405% and 349% as compared to the measurement of the control.

**Figure 9:**
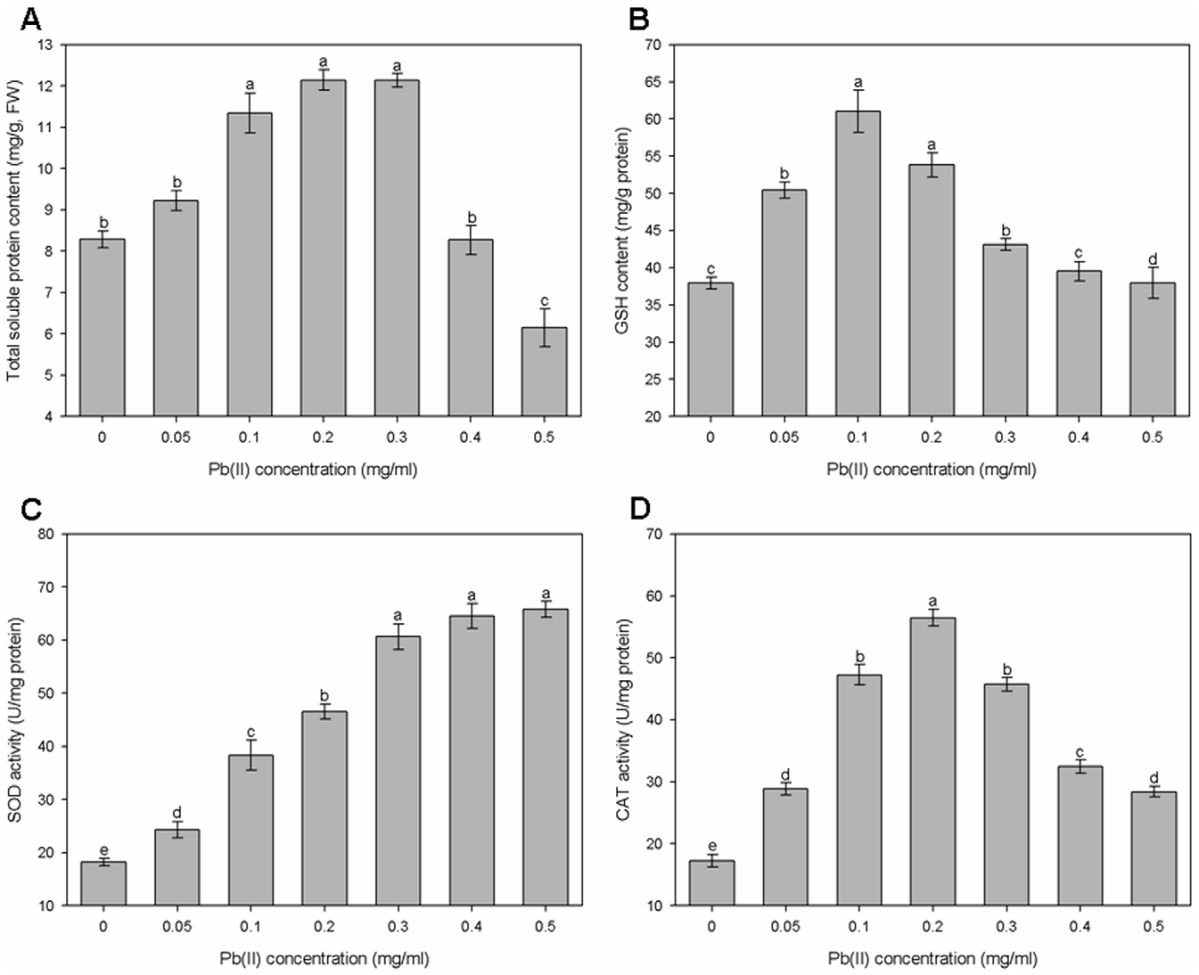
The responses of antioxidant substances to the toxicity of Pb(II). The contents of total soluble protein (A) and GSH (B) and the activities of SOD (C) and CAT (D) in the hyphae of *G*. *cylindrosporus* cultivated for a week in liquid MMN medium supplemented with a series of increasing Pb(II). FW represented fresh weight. Different letters above bars indicate significant differences (P<0.05) assessed by Duncan's test. Data are means ± SE (n = 4).

Changes in the soluble protein content in *G*. *cylindrosporus* under different concentrations of Pb(II) are shown in Fig. 9A, which indicates that the addition of 0.05 to 0.3 mg/ml Pb(II) to the medium gradually increased the total soluble protein content. Higher Pb(II) concentrations, especially the concentration of 0.5 mg/ml, decreased the protein content. Like the changes in the contents of melanin and total soluble protein, GSH content in *G*. *cylindrosporus* greatly increased by treatment with low concentrations of Pb(II) (Fig. 9B). When the concentration of Pb(II) increased to 0.1 mg/ml, GSH content reached a maximum value of 0.69 mg/g. Beyond this concentration, progressive decrease of GSH content occurred as shown in Fig. 9B. Unlike the other antioxidant substances, SOD activity in the hyphae of *G*. *cylindrosporus* showed a significant positive correlation with Pb(II) concentrations in the medium (r = 0.957, P<0.001) (Fig. 9C). The initial increase in CAT activity, however, declined after reaching a maximum value at the concentration of 0.2 mg/ml (Fig. 9D).

## Discussion

In this work, we found that five DSE species isolated from the roots of *A*. *adsurgens* on lead-zinc mine tailings belong to different taxa based on the sequences data of the ITS1-5.8S-ITS2 (ITS) regions of the nuclear rDNA and morphological characteristics. Resynthesis experiments of the five isolates association with seedlings of *A*. *adsurgens* demonstrated that they are typical DSE fungi. Melanized septate hyphae and microsclerotia were observed in the root cortex using conventional staining methods for fungi (Fig. 3). In addition, some of the species that we isolated had either been recognized as DSE by other researchers or belong to the same genus as typical DSE. Addy et al. [42] insisted that *G*. *cylindrosporus*, the teleomorph of *Phialophora graminicola*, was a typical DSE. Zhang et al. [43] reported that the sequence of rDNA ITS regions of DSE fungi LBF-2 had 96% similarity as compared to the GenBank sequence of *P*. *chrysanthemicola*. Yuan et al. [44] found that *C*. *cladosporioides*, isolated from roots of *Artemisia annua*, was characterized with dark pigmented hypha and sclerotium-like structures. The genera of *Phialophora* and *Exophiala* as DSE harboring in host roots were also reported [12], [45].

It has been demonstrated that the fungal ecotypes from heavy metal contaminated sites are more tolerant to heavy metals than reference strains from uncontaminated sites [46], [47]. However, toxic symptoms may vary widely between different fungi and for different metal species [14]. EC_50_ and MIC were selected to measure the sensitivity of these isolates to Pb(II), Zn(II), and Cu(II), which have been widely used for evaluation of heavy metal tolerance of filamentous fungi [12], [48]. In our work, *G*. *cylindrosporus* was selected for the further research because of its remarkable tolerance to Pb(II). The biomass and colony diameter of *G*. *cylindrosporus* exceeded the control at low concentrations of Pb(II). Pb(II) appeared less toxic for *G*. *cylindrosporus* in comparison to the other metals.

Mechanisms of toxic metal tolerance in fungi include reduction of metal uptake and/or increased efflux, metal immobilization, e.g. cell-wall adsorption, extracellular precipitation, extracellular binding by polysaccharides and extracellular metabolites, intracellular compartmentalization and intracellular sequestration as metallothioneins and phytochelatins, etc [4], [14], [49]. However, different species may have individual strategies to reduce toxicity of heavy metals. Morselt et al. [50] found that tolerance to heavy metals in the ectomycorrhizal fungus *Pisolithus tinctorius* is based on the presence of metallothionein-like proteins. Cánovas et al. [51] reported that GSH in *Aspergillus* sp. P37 was found to be responsible for the binding of arsenic. Baldrian [52] also proposed that the intact cell walls of many fungal species exhibit high binding capacities for heavy metals. Melanins, located in and/or exterior to cell walls, could reduce the toxicity of Cu, Zn, Cd, and Pb [53]. Zhang et al. [12] indicated that the mechanism of heavy metal tolerance of DSE strains isolated from metal soil would be a complex process, and they found that SOD and CAT activities in the hyphae of *E*. *pisciphila* had positive correlations with Pb(II) and Cd(II) concentrations.

Heavy metals exert toxic effects in many ways, and morphogenesis of fungal colonies and hyphae could be influenced by the presence of heavy metals. In our study, there were remarkable changes in colony morphology of *G*. *cylindrosporus* under Pb(II) stress as compared to the heavy metal-free control. The colony color turned dark or brown on agar medium under Pb(II) concentrations of 0.2 mg/ml, 0.6 mg/ml, and 1.0 mg/ml (Fig. 5B, C, and D). Color changes of mycelia exposed to metals may be an indicator of metal complexation [54]. This kind of change has also been reported by other researchers [55], [56]. The reasons for color changes of colonies or mycelia are complex. However, in our study, melanin may be the primary component that leads to blackening of colonies. Similar results were reported by Guillén and Machuca [57] and Gadd [14]. They indicated that melanin is responsible for the color changes in the mycelia, and have been related to survival mechanisms that alleviate the metals toxicity. Changes in mycelial morphology due to toxicity of certain metals are frequent in the majority of fungi [57]. For example, in the ectomycorrhizal fungus *Pleurotus ostreatus*, the addition of Cd led to an increase in hyphal density caused by increased number of laterals per branch point and a decrease in the distance between branch points [58]. Lanfranco et al. [59] found that high concentrations of zinc altered hyphal morphology and that the most characteristic features of zinc-treated hyphae were the overall increases in hyphal branching, swelling, and septation. However, different species may have different changes in mycelial morphology under heavy metal ions stress. In this work, we found that there was twisting of individual hyphae, formation of intertwined hyphal strands, and looping of the individual hyphae when *G*. *cylindrosporus* was cultured on solid medium under Pb(II) stress (Fig. 6). Similar observation was also made by Lilly et al. [17]. This unusual mycelial morphology may be explained by metal ions affecting the mechanisms responsible for maintenance of electrochemical gradients across the apex, such as the H^+^-ATPase and nutrient channels [60], [61]. Changes in mycelial morphology in response to toxic metals may be closely related to the nutritional status of the habitat or growth medium [61]. Nutritional components will be destroyed with the addition of toxic heavy metals into medium, which may be one important reason for the changes. There were also significant changes in hyphal morphology, but with distinctive characters, when *G*. *cylindrosporus* was cultured in liquid medium supplemented with different concentrations of Pb(II). Hyphae curled and twisted when cultivated in liquid medium with 0.1 mg/ml of Pb(II) (Fig. 7B). The presence of high concentrations of Pb(II) caused new variation of hyphal morphology, and the most characteristic features, as compared to the characteristic features of the control, were hyphal swelling with irregularly thickened cell walls (Fig. 7C and D). We believe that these changes have a close relationship with the variation in cell wall components. The increased melanin content detected in hyphae treated with Pb(II) may indicate that melanin, together with other cell wall components, contributed to the fungal response to the increase of metal concentrations in the medium.

These DSE species, isolated from heavy metal contaminated soil, have developed several strategies of defense against toxic metals. The contents of melanin and GSH and the activities of SOD and CAT in *G*. *cylindrosporus* were examined to detect the responses of antioxidant substances to Pb(II) stress. Melanin possesses many potential sites for metal binding. Therefore, it has high capacities for metal biosorption, with the majority of metal remaining within the wall structure [62]. A variety of heavy metals may induce or accelerate the production of melanin pigmentation in certain fungi [13], [63]. In this study, we found that melanin content in *G*. *cylindrosporus* obviously increased when exposed to 0.2 and 0.3 mg/ml Pb(II) and decreased slightly at two higher concentrations. The toxic effect of heavy metals appears to be related to production of ROS and the resulting unbalanced cellular redox status. ROS may cause wide-ranging damage to proteins, nucleic acids, and lipids, eventually leading to cell death [52], [64]. Fungi produce antioxidant molecules and activate enzymes (e.g. GSH, SOD, and CAT) to remove oxygen radicals and their products and/or repair oxidative damage [65]. GSH has been reported as the major heavy metal-responsive thiol, playing an important role in cellular protection during oxidative stress [66]. In our study, GSH content increased at low concentrations of Pb(II). A similar finding has been reported by Ott et al. [67] and Hegedüs et al. [48]. SOD and CAT are crucial for cellular detoxification, controlling the levels of superoxide anion radical and hydrogen peroxide [66], [68]. We found that SOD activity in *G*. *cylindrosporus* was positively correlated with Pb(II) concentrations, and gradually increased within the range of concentrations of Pb(II) we selected (Fig. 9C). The enhanced synthesis of SOD, whose function was to scavenge ROS, suggested that oxidative stress had a great influence on the growth of *G*. *cylindrosporus* in the presence of Pb(II). These results are in accordance with the data of Todorova et al. [69] for SOD production by *Aspergillus niger* B77 in the presence of cadmium. The CAT activity declined when Pb(II) concentration was above 0.2 mg/ml, but a strong activation of CAT was observed under Pb(II) stress at the low concentrations tested (Fig. 9D). It could be assumed that the enhanced levels of SOD were insufficient to remove the generated ROS completely, which are capable of provoking the inhibition of CAT [69]. Nevertheless, CAT activity at the high concentrations still increased as compared to the CAT activity of the control. Our findings point out that SOD and CAT enzymes play an effective role in protecting *G*. *cylindrosporus* against oxidative stress induced by Pb(II), but SOD appears to act as the primary defense against acute Pb(II) stress.

Mechanisms of heavy metal tolerance in DSE fungi are exceedingly complex; therefore, further studies are necessary. Since DSE fungi are typical root endophytes, their potential effects on host plants should be considered. DSE fungi can affect the heavy metal uptake of their host plants, and enhance the tolerance of their host plants to heavy metal stress. However, little is known about whether the alleviation of metal phytotoxicity can be attributed primarily to DSE fungi or to the result of the interactions between DSE fungi and host plants. The mechanisms by which DSE fungi help their hosts enhance the heavy metal tolerance may be more meaningful than their mechanisms for self protection. Future research should be focused on this mutually beneficial relationship between DSE fungi and their hosts. Furthermore, their ecological function in stressed ecosystems deserves our attention.
